# Treatment of conjunctival papilloma with topical interferon alpha-2b - case report

**DOI:** 10.1097/MD.0000000000019181

**Published:** 2020-02-14

**Authors:** Bartłomiej Bolek, Adam Wylęgała, Sławomir Teper, Joanna Kokot, Edward Wylęgała

**Affiliations:** Chair and Clinical Department of Ophthalmology, School of Medicine in Zabrze, Medical University of Silesia in Katowice, District Railway Hospital, Katowice, Poland.

**Keywords:** conjunctival diseases, conjunctival papilloma, interferon alpha-2b

## Abstract

**Rationale:**

Conjunctival papilloma is a benign neoplastic lesion of epithelial origin, with a minimal tendency toward malignancy and an exophytic growth type. Etiological factors that contribute to the appearance of papilloma are UV exposure, HPV infection, smoking, and immunodeficiency. A case report of limbal conjunctival papilloma treatment with topical interferon alpha-2b eye drops applied to the conjunctival sac.

**Patient concerns:**

A 49-year-old female patient treated in the Clinical Department of Ophthalmology for conjunctival lesion located in the temporal quadrant near the limbus of the right eye. Ocular examination of the patient's eye did not show any abnormalities: visual acuity—20/20, intraocular pressure—16 mmHg.

**Diagnoses:**

During physical examination, a broad-based pink lesion of size 4 mm × 6 mm was found in interpalpebral bulbar conjunctiva with prominent feeder vessels and soft consistency. Lesion in the biomicroscopic examination did not show any sign of malignancy. In Swept Source Optical Coherence Tomography (SS-OCT) there was no sign of infiltration into sclera or cornea. In vivo confocal microscopy (IVCM) examination, revealed loss of the normal conjunctival epithelium - hyper-reflective cells, variation of cell size. A clinical diagnosis of limbal conjunctival papilloma was made.

**Interventions:**

Interferon alpha-2b 1 million IU/ml eye drops were applied 4 times a day for 5 months. The treatment lasted 5 months without changing the dosing regimen. Three months after the start of the interferon treatment, a complete regression of the lesion was achieved. Treatment was continued for the following 2 months. Controlled IVCM after 6 months showed only few hyper-reflective cells and fibrotic tissue.

**Outcomes:**

Treatment of conjunctival papilloma with topical interferon alpha-2b led to the complete regression of the lesion. Although after the therapy the conjunctiva appears normal both in slit lamp and in the OCT examination, there is a noticeable fibrosis of the tissue in confocal microscopy. During the 14-month follow-up period, there were no recurrent lesions.

**Lessons:**

In this study, topical interferon alpha-2b has been shown to be an effective and safe therapy for small-to-medium-size conjunctival papilloma without any sign of malignancy.

## 1 Introduction

Conjunctival papilloma is a benign neoplastic lesion of epithelial origin, with a minimal tendency toward malignancy and an exophytic growth type.^[1]^ Etiological factors that contribute to the appearance of papilloma are UV exposure, HPV infection, smoking, and immunodeficiency.^[2]^ Based on the location, age of occurrence, tendency to regrow, and histopathology, conjunctival papilloma can be categorized into the following types: squamous, limbal, and inverted.^[1]^ Most of these lesions were located medially (89%) and inferiorly (71%).^[1]^ To be more precise about the most common location, authors are not unanimous—bulbar conjunctiva (42%—Ash),^[3]^ palpebral conjunctiva (38%—Sjö et al),^[1]^ and caruncle (23%—Kaliki et al)^[4]^ Excision is commonly used method to treat this type of lesions. However it does not always produce the expected result due to frequent recurrences. In cases of small or recurrent lesions pharmacotherapy can be used as the first choice. Regression of the lesion can be achieved with topical interferon alpha-2b.

## 2 Case report

**Figure 1:**
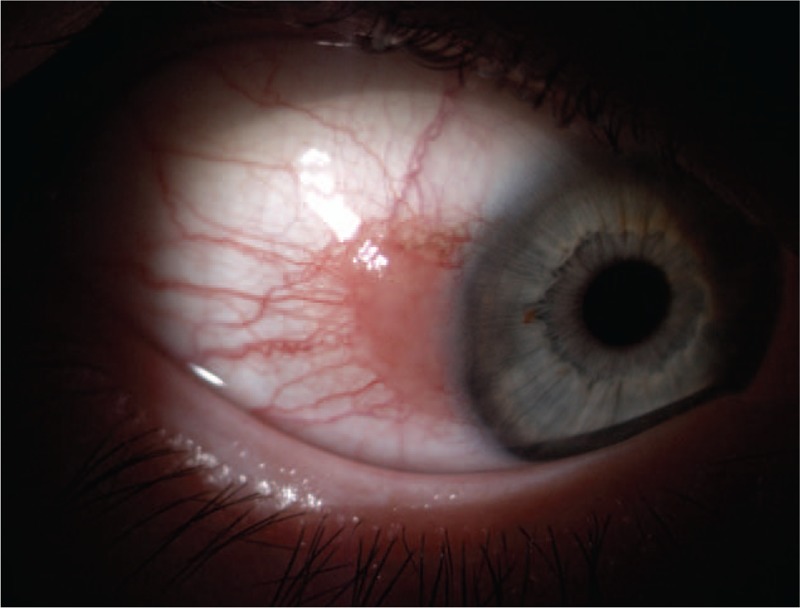
Slit lamp photography showed broad-based pink lesion of a limbal conjunctival papilloma prior to the start of treatment.

**Figure 2:**
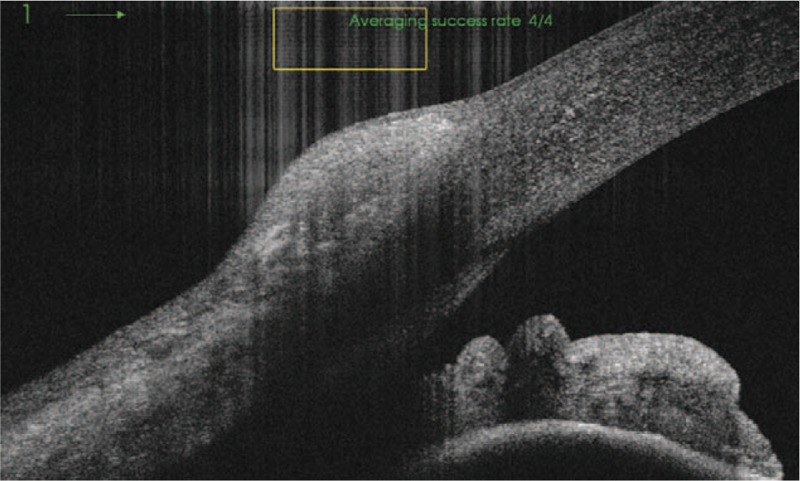
SS-OCT scan of a limbal conjunctival papilloma —no sign of infiltration into sclera or cornea.

**Figure 3:**
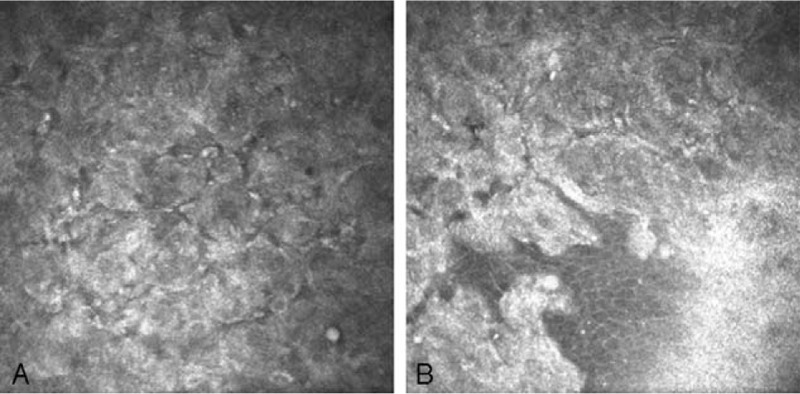
Confocal microscopy of limbal conjunctival papilloma-revealed loss of the normal conjunctival epithelium structure – and presence of hyper-reflective cells (A, B), variation of cell size (A,B).

**Figure 4:**
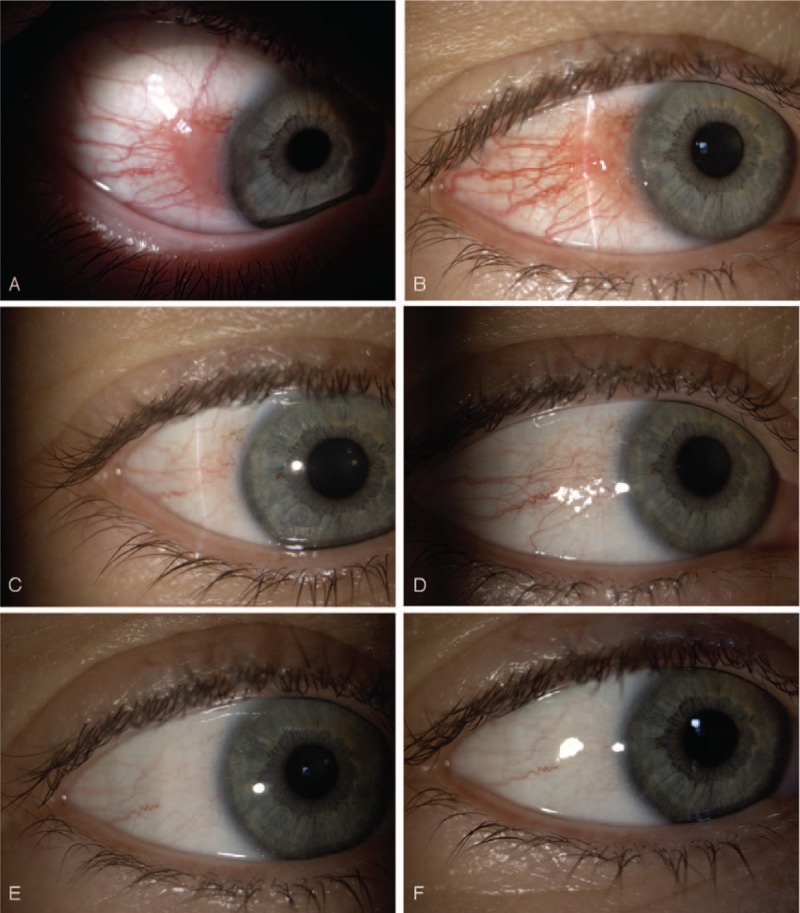
Treatment outcome of limbal conjunctival papilloma. Top right shows the ocular surface before the therapy (A). Treatment process after 1 month (B), 3 month (C), 5 month (D), 6 month (E). The eye showed significant improvement, no signs of papilloma was noted at 14 month follow up (F).

**Figure 5:**
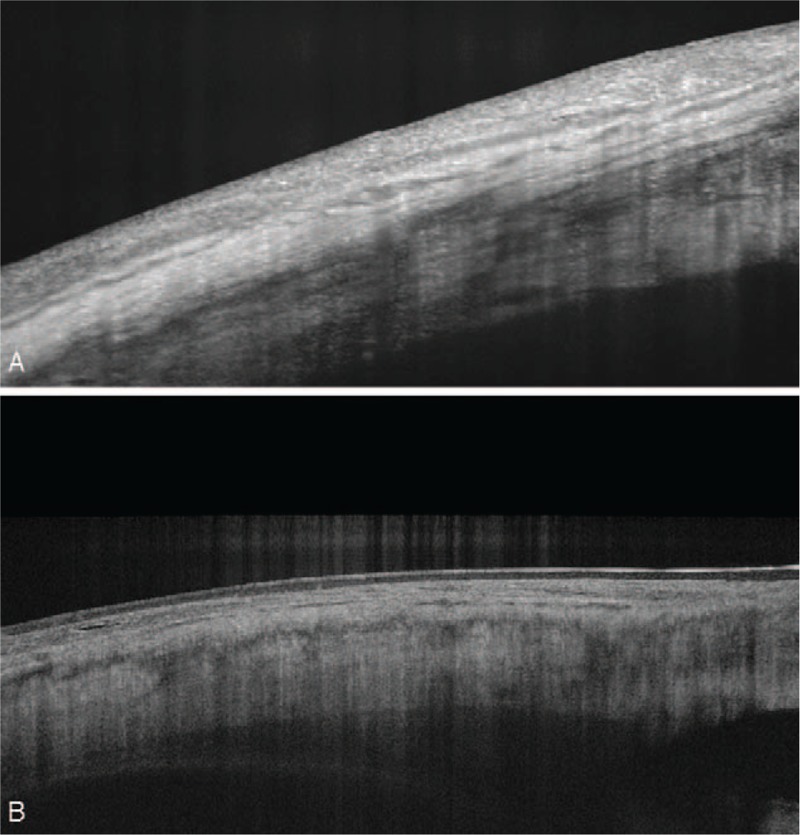
SS-OCT scan of limbal conjunctival papilloma. Treatment process after 3 month (A) and 14 months (B).

**Figure 6:**
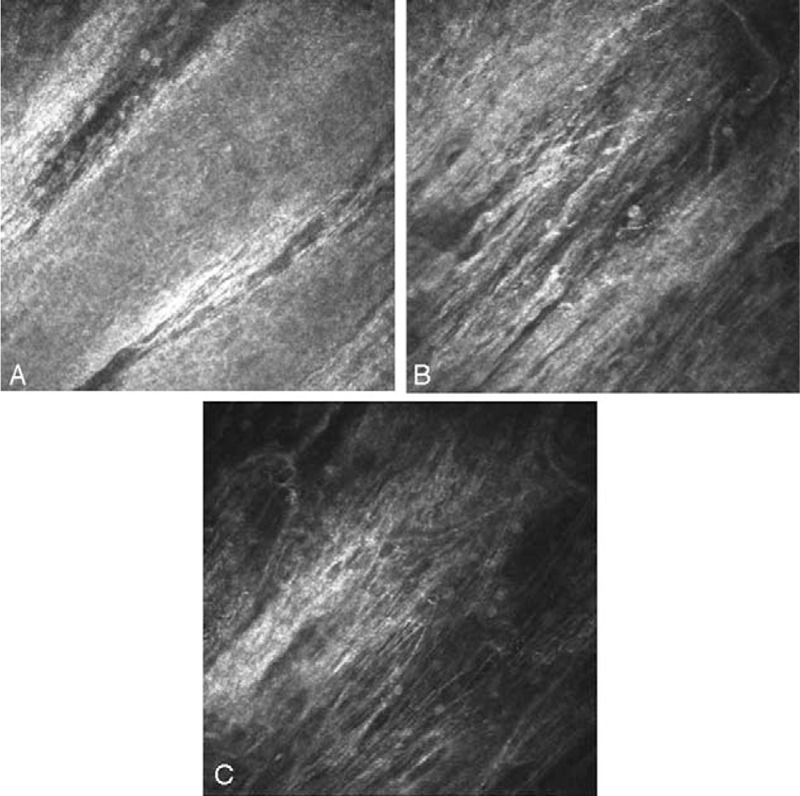
Confocal microscopy of limbal conjunctival papilloma—6 months after treatment presented normal epithelium, few hyper-reflective cells and fibrotic tissue.

A 49-year-old female patient was treated in the Clinical Department of Ophthalmology for conjunctival lesion located in the temporal quadrant near the limbus of the right eye. During physical examination, a broad-based pink lesion of size 4 mm × 6 mm was found in interpalpebral bulbar conjunctiva with prominent feeder vessels and soft consistency (Fig. 1). Lesion in the biomicroscopic examination did not show any sign of malignancy. Moreover, ocular examination of the patient's eye did not show any abnormalities: visual acuity—20/20, intraocular pressure—16 mmHg. In SS-OCT (DRI OCT Triton with anterior attachment), there was no sign of infiltration into sclera or cornea (Fig. 2). In vivo confocal microscopy (IVCM Rostock Corneal Module, Heidelberg Engineering GmbH, Dossenheim, Germany) examination, revealed loss of the normal conjunctival epithelium - hyper-reflective cells, variation of cell size (Fig. 3). After above examinations a clinical diagnosis of limbal conjunctival papilloma was made and interferon alpha-2b 1 million IU/ml eye drops were applied 4 times a day for 5 months. Since the beginning of the treatment, the patient was monitored at the clinic after 3 weeks and then at every 6-week interval. The treatment lasted 5 months without changing the dosing regimen. Three months after the start of the interferon treatment, a complete regression of the lesion was achieved (Figs. 4 and 5). Treatment was continued for the following 2 months. Besides itching, which occurred at the beginning of the treatment, there was no other side effect of topical interferon. Visual acuity remained unchanged. Controlled IVCM was performed after 6 months from the initiation of therapy, showed only few hyper-reflective cells and fibrotic tissue (Fig. 6).

Treatment of conjunctival papilloma with topical interferon alpha-2b led to the complete regression of the lesion. Although after the therapy the conjunctiva appears normal both in slit lamp and in the OCT examination, there is a noticeable fibrosis of the tissue in confocal microscopy. During the 14-month follow-up period, there were no recurrent lesions.

## 3 Discussion

Squamous cell papilloma appears as a grayish red, fleshy, soft, pedunculated, or raised mass with an irregular surface located in the inferior fornix, caruncle, and palpebral regions. It is strongly associated with HPV^[5]^ (mostly type HPV11).^[6,7]^ Other types of virus have also been found: type 6 (group with low oncogenic potential—benign lesion)^[8]^ and type 16, 18, or 45 (group with high oncogenic potential—associated with the development of squamous cell neoplasia or conjunctival squamous cell carcinoma).^[5,7,9,10]^ It occurs mainly in children and adults under 20 years of age. This type of papilloma has a high recurrence rate.

Limbal conjunctival papillomas are sessile, gelatinous lesions with epithelium dysplasia and feeder vessels.^[4]^ This type of lesions arises from UV radiation exposure rather than due to infectious factors. In comparison to squamous papilloma, it has a lower recurrence rate. The former usually does not cause deterioration or loss of vision, while the latter may contribute to loss of vision when it extends into the visual axis.

These lesions rarely become malignant^[1]^—signs of which include local inflammation, keratosis, adhesions between the tarsal and bulbar conjunctiva and characteristic localization^[2]^. Majority of the conjunctival squamous cell carcinomas are in the interpalpebral zone near to the limbus. The papilloma should be differentiated from ichthyosis, sebaceous gland cancer, conjunctival lymphoma, or squamous cell neoplasia, including conjunctival squamous cell carcinoma. Unfortunately, there are only contradicting reports about the use of IVCM to distinguish between malignant and benign conjunctival lesions.^[11–13]^

Currently, there are several methods available for the effective treatment of conjunctival papilloma, including excision, cryotherapy, CO_2_ laser therapy, and pharmacotherapy along with the use of cytotoxic and immunomodulating medication. Small papillomas may regress spontaneously.^[1]^ Excision of the lesion does not always produce the expected result due to frequent recurrences (from 6% to 27%^[1]^) and possible seeding, which may lead to multiple new papillomas. Recurrences are more common in children and adolescents than in adults.^[1]^ Cryotherapy, however, is characterized by lower recurrence rate and reduced scarring compared with conventional excision.^[4]^ In the case of lesions without malignant features, recurrence tendency, pharmacotherapy appears to be a better alternative compared with invasive methods. In the literature, there are several reports on the effective use of topical H_2_ receptor antagonists (cimetidine),^[14,15]^ dinitrochlorobenzene,^[16]^ interferon alpha-2b,^[4,17–22]^ mitomycin C,^[23,24]^ and bevacizumab^[25]^ for the treatment of such types of disorders. According to some authors, surgical excision of the lesion and cryotherapy to the adjacent conjunctiva are the treatment of choice among patients.^[2]^ Adjuvant oral cimetidine and/or topical interferon alpha-2b reduce the recurrence rate, which seems to be the proper approach considering large lesions.^[4]^ Taking into consideration the cytotoxicity of mitomycin C (dry eye syndrome, corneal melt, punctual stenosis)^[2]^ and the relatively long period for interferon to achieve the clinical effect,^[18]^ despite there is only one report, it is worth to look at bevacizumab as adjuvant therapy for the effective treatment of recurrent lesions. In our patient, complete regression of the lesion is similar result to other reports regarding topical interferon alpha-2b treatment in conjunctival papilloma.^[18,19,21,22]^ In cases of small lesions or in recurrent papillomas, pharmacotherapy^[19]^ can be used as the first choice. Currently, there are no available reports in the literature comparing the effectiveness of aforementioned methods.

## Author contributions

**Conceptualization:** Sławomir Teper, Edward Wylęgała.

**Data curation:** Bartlomiej Bolek, Edward Wylęgała.

**Formal analysis:** Bartlomiej Bolek, Adam Wylęgała.

**Investigation:** Bartlomiej Bolek, Sławomir Teper, Joanna Kokot.

**Methodology:** Bartlomiej Bolek, Adam Wylęgała, Sławomir Teper, Joanna Kokot.

**Project administration:** Bartlomiej Bolek, Adam Wylęgała, Sławomir Teper, Edward Wylęgała.

**Resources:** Sławomir Teper.

**Software:** Bartlomiej Bolek, Joanna Kokot.

**Supervision:** Edward Wylęgała.

**Validation:** Edward Wylęgała.

**Visualization:** Bartlomiej Bolek, Joanna Kokot.

**Writing–original draft:** Bartlomiej Bolek, Joanna Kokot.

**Writing–review & editing:** Bartlomiej Bolek, Adam Wylęgała, Sławomir Teper, Joanna Kokot, Edward Wylęgała.
